# Enrichment of Whole-Grain Breads with Food-Grade Extracted Apple Pomace Bioactives Enhanced Their Anti-Inflammatory, Antithrombotic and Anti-Oxidant Functional Properties

**DOI:** 10.3390/antiox13020225

**Published:** 2024-02-11

**Authors:** Alexandros Tsoupras, Donal Moran, Katie Shiels, Sushanta Kumar Saha, Ibrahim M. Abu-Reidah, Raymond H. Thomas, Shane Redfern

**Affiliations:** 1 Hephaestus Laboratory, Department of Chemistry, International Hellenic University, 65404 Kavala, Greece; 2 Department of Biological Sciences, University of Limerick, V94 T9PX Limerick, Ireland; d.moran160@gmail.com; 3 Shannon Applied Biotechnology Center, Technological University of the Shannon: Midlands Midwest, Moylish Park, V94 E8YF Limerick, Ireland; katie.shiels@tus.ie (K.S.); sushanta.saha@tus.ie (S.K.S.); srshaneredfern6@gmail.com (S.R.); 4 School of Science and the Environment, Memorial University of Newfoundland, Corner Brook, NL A2H 5G4, Canada; iabureidah@mun.ca; 5 Biotron Experimental Climate Change Research Centre, Department of Biology, Western University, London, ON N6A 5B7, Canada; rthoma2@uwo.ca

**Keywords:** apple pomace, whole-grain bread, bioactives, polar lipids, phenolics, carotenoids, PAF, anti-inflammatory, antithrombotic, antioxidant

## Abstract

Apple pomace (AP) is a bio-waste product of apples that is co-produced as a by-product during apples’ processing for making apple-based products, mainly apple juice, cider and vinegar. AP is a rich source of several bioactives that can be valorized as ingredients for developing novel functional foods, supplements and nutraceuticals. Within the present study, food-grade extracts from AP with different tannin contents were found to contain bioactive polar lipids (PLs), phenolics and carotenoids with strong anti-oxidant, antithrombotic and anti-inflammatory properties. The extract from the low-in-tannins AP showed stronger anti-inflammatory potency in human platelets against the potent thrombo-inflammatory mediator platelet-activating factor (PAF), while it also exhibited considerable anti-platelet effects against the standard platelet agonist, adenosine diphosphate (ADP). The infusion of 0.5–1.0 g of this bioactive AP extract as functional ingredients for whole-grain bread-making resulted in the production of novel bio-functional bread products with stronger anti-oxidant, antithrombotic and anti-inflammatory potency against both PAF and ADP in human platelets, compared to the standard non-infused control breads. Structural analysis by LCMS showed that the PL-bioactives from all these sources (AP and the bio-functional breads) are rich in bioactive unsaturated fatty acids (UFA), especially in the omega-9 oleic acid (OA; 18:1n9), the omega-3 alpha linolenic acid (ALA; 18:n3) and the omega-6 linoleic acid (LA; 18:2n6), which further supports their strong anti-inflammatory and antithrombotic properties. All food-grade extracted AP including that infused with AP-bioactives novel functional breads showed higher hydrophilic, lipophilic and total phenolic content, as well as total carotenoid content, and subsequently stronger antioxidant capacity. These results showed the potential of appropriately valorizing AP-extracts in developing novel bio-functional bakery products, as well as in other health-promoting applications. Nevertheless, more studies are needed to fully elucidate and/or validate the anti-inflammatory, antithrombotic and antioxidant potential of novel bio-functional products across the food and cosmetic sectors when infused with these AP bioactives.

## 1. Introduction

Oxidative stress, thrombotic and inflammatory complications have been associated with several chronic disorders. According to the World Health Organization (WHO), worldwide three out of five people die due to chronic inflammatory non-communicable diseases such as stroke, chronic respiratory diseases, heart disorders, cancer, obesity and diabetes. Subsequently, chronic inflammatory disease has been ranked as the greatest threat to human health [1]. The onset and progression of such inflammation-related chronic disorders due to chronic inflammation involves a continuous and inappropriate inflammatory activation and the cell–cell interaction of platelets and leukocytes, followed by oxidative damage and endothelial dysfunction at the inflamed sites, in which reactive oxygen species (ROS) and thrombo-inflammatory mediators, such as platelet-activating factor (PAF) and thrombin, as well as several classic platelet agonists, such as adenosine diphosphate (ADP) and collagen, are usually implicated [2].

Dietary bioactives from sustainable sources, such as those present in health fruits and in beverages from these fruits, with anti-inflammatory and antioxidant effects can protect against inflammatory and oxidative manifestations involved in these pathologies [3,4,5,6,7,8]. Moreover, antioxidants and anti-inflammatory phenolics and lipid bioactives, such as several bio-functional dietary polar lipids (PLs) present in the by-products of fruit processing, have also shown significant potential against several oxidative, thrombotic and inflammatory pathways involved in the onset and development of chronic disorders [5,6,9,10]. Subsequently, the dietary bioactives of fruits’ by-products have also gained interest as sustainable sources of natural dietary bioactives that can be valorized as ingredients in functional foods and food supplements or nutraceuticals with anti-inflammatory health-promoting properties, in a circular economy design [5,6,9,10]. 

According to the food and agriculture organization of the United Nations (FAOSTAT), apple is one of the five fruit species accounting for the huge increase in world fruit production since 2000 [11], as it is one of the most highly preferable fruits for consumers worldwide, due to its nutritional value and accessibility throughout the year. The worldwide apple industry produces several products such as juice, cider and vinegar with low lipid contents. Nevertheless, it was recently found that both the fruit and its apple juice and cider products contain bioactive PLs with similar anti-inflammatory and anti-platelet properties observed in other beverages derived from the fermentation of fruits’ juices [5,6,7,8,9]. During apple processing into its products, a large amount of apple pomace (AP) as an apple by-product is produced. This leads to approximately 70 million tons per annum of AP globally, which, due to its nutritional content, can be utilized as a source of bioactives and nutrients in the development or formulation of different functional food products [9,12,13,14]. 

The dietary content of AP is mainly composed of carbohydrates, protein, lipids, fiber and ash [9]. These macro-phytonutrients are found in both the skin and the flesh (95%) [9,12]. Apple is also a source of phytochemicals like phenolic compounds, such as several flavonoids, which are also found in high amounts in AP too [9,14]. During processing, many of the hydrophilic water soluble components of these nutrients and bioactive compounds are transferred in the watery juice product. However, those remaining within AP flesh and skin also contain valuable amounts of phenolic compounds [9], as well as other bioactives, similarly to other fruits’ derived pomaces, such as grapes and their grape pomace, in which bioactives with anti-inflammatory potency against PAF-related inflammatory manifestations and the subsequent oxidation of LDL-cholesterol have been reported [5,9].

Even though some hydrophilic phenolic and nucleotide-based compounds found in apples have shown significant bioactivities in general [14,15,16,17,18,19,20], still they were not found to inhibit PAF or ADP-induced platelet aggregation and thrombo-inflammation [7,8,21,22]. 

In contrast, several amphiphilic/lipid bioactive compounds with anti-inflammatory potency have recently been found in both apple products (apple juice and cider) [7,8] and by-products (AP) [22]. More specifically, both apple juice and cider were found to contain several subclasses of PLs bioactives in the amphiphilic extracts, including phosphatidylcholines (PCs) and phosphatidylethanolamines (PEs), which exhibited the most potent anti-platelet and anti-inflammatory effects against both ADP and PAF in human platelets [7]. The utilization of the t most resilient to fermentation bioactive apple must yeast strains, has shown to facilitate the production of more bioactive functional apple cider containing PL bioactives with strong anti-inflammatory, anti-platelet and cardio-protective potency [8]. 

Moreover, the apple cider by-products (ACBP) of apple varieties that are usually used in juice/cider production have not yet been thoroughly studied for their content in amphiphilic/lipid bioactives that possess anti-oxidant, anti-inflammatory and anti-platelet benefits. Thus, an evaluation for these apple by-products, as sustainable sources of amphiphilic/lipid and phenolic bioactives, is needed. A preliminary study of our group has indicated that in apple pomace, there are plenty of amphiphilic phenolics and PL bioactives with strong anti-inflammatory and anti-platelet properties [22]. However, these were extracted with conventional extraction solvents and a methodology based on chloroform, methanol and petroleum ether, which are not green, food-grade solvents. 

Thus, within the present study, the food-grade extracts of several ACBP (with different tannin contents) that are rich in polar lipid bioactives (ACBP-FGE-PLs) that also contain some amphiphilic phenolics were assessed for putative anti-oxidant, anti-platelet and anti-inflammatory activities. Thereafter, the most bioactive ACBP-FGE-PLs extract was further utilized as a functional ingredient for the development of novel functional breads (whole grain), and the improved anti-inflammatory, antithrombotic and antioxidant health-promoting properties of the functional products derived were assessed.

## 2. Materials and Methods

### 2.1. Materials—Instrumentation

Solvents of analytical-grade reagents and other consumables were obtained from Fisher Scientific Ltd. (Dublin, Ireland). For blood sampling, both 20G safety needles and evacuated sodium citrate S-monovettes were obtained from Sarstedt Ltd. (Wexford, Ireland), while standard BSA, PAF, thrombin and egg phospholipids were purchased from Sigma Aldrich (Wicklow, Ireland). All the other consumables related to platelet aggregation assays were purchased from Labmedics LLP (Abingdon on Thames, UK). An Eppendorf 5702R centrifuge (Eppendorf Ltd., Stevenage, UK) was utilized for all centrifugations of blood samples, while all platelet aggregation bioassays were performed on a Chronolog-490 two-channel turbidimetric platelet aggregometer (Havertown, PA, USA), coupled to the accompanying AGGRO/LINK software package. Spectrophotometric analysis and absorbance measurements for the evaluation of total phenolic content (TPC), total antioxidant activity (TAA) and total carotenoid content (TCC) were carried out using a Cytation imaging microplate reader (BioTek, Winooski, VT, USA).

### 2.2. Extractions of ACBP Bioactives and Functional Breads

Apple pomace samples were obtained from Aston Bitter (high content in tannins), Dabinett (intermediate content in tannins) and Jonagold (low content in tannins) apple varieties, which were all grown according to normal commercial practice on the plantation of the “Con Traas’s Apple Farm” located in County Tipperary, Ireland. The apples of these three varieties were washed and pressed at a pressing machine while the produced juice was further processed for the production of commercial juice and cider. From the remaining ACBP co-produced after this stage, also known as the “apple pomace”, which comprises the core, exhausted soft tissue, peel, stems and seeds, the samples were provided, while the rest was further processed for fertilizer uses. 

The ACBP-FGE-PLs extracts were obtained from three samples (n = 3) of 100 g from each one of these three ACBP types (low, intermediate and high in tannins), according to Invention Disclosure: IDF 2006504, Technology Transfer Office of the University of Limerick, 2021, Ireland. Briefly, each sample was homogenized mechanically using a Waring blender (Fisher Scientific Ltd., Dublin, Ireland) in the presence of the appropriate food-grade HPLC-grade solvents, following EU legislations on extraction solvents used in the production of foodstuffs and food ingredients [23,24]. Then, the obtained ACBP-FGE-PLs extract was filtrated from the precipitated remnants with filtering papers of 110 mm (Whatman, Maidstone, UK) under vacuum conditions by pumping in a Buchner-based filtering device. Solvents were evaporated from the samples using flash rotary evaporation (Buchi Rotavapor, Mason Technology, Dublin, Ireland), and the extract was transferred into small pre-weighed glass vials, where all the remaining solvents were further evaporated under a stream of nitrogen. The acquired ACBP-FGE-PLs extracts were weighed and stored under a nitrogen atmosphere in −20 °C for further analysis.

A total of 0.5–1.0 g of the most bioactive ACBP-FGE-PLs extract was further utilized as a functional ingredient in whole-grain breads, according to Invention Disclosure: IDF 2006504, Technology Transfer Office of the University of Limerick, 2021, Ireland. More specifically, the control breads contained no apple pomace lipid extract, the low-concentration breads (LB) contained ~0.15 g ACBP-FGE-PLs, the intermediate-concentration breads (IB) contained ~0.5 g ACBP-FGE-PLs and the high-concentration breads (HB) contained ~1 g of ACBP-FGE-PLs, respectively. Apple pomace low in tannins was used in these breads as they were previously recorded to contain the greatest bioactivity in comparison to the medium and high levels in tannin apple pomace in the food-grade extracts, in comparison to their relative conventional extracted PL as previously reported by Tsoupras et al. [22].

The total lipids (TLs) of 100 g bread samples from batches of each type of the produced functional breads that were infused with different concentrations of ACBP-FGE-PLs extracts (LB, IB and HB; n3 in each case), as well as from batches of the control breads (n = 3, in this case, too) that did not contain any ACBP-FGE-PLs as ingredients, were obtained by extractions based on the Bligh and Dyer method [25], while these bread-derived TLs were further separated into neutral lipids (NLs) and PLs by counter-current distribution based on the Galanos and Kapoulas methodology [26], as previously described [7,22]. More specifically, TL extractions were performed in breads just after they were cooled down and no more than 1 h after their production in the oven, as previously described [7,22], by homogenizing each bread sample in a chloroform/methanol/water (1:2:0.8; *v*/*v*/*v*) monophasic system, followed by a filtration of the extracted lipids from the precipitated remnants by pumping under vacuum conditions in a Buchner-based filtering device with filtering paper of 110 mm (Whatman, Maidstone, UK). This filtrate was transferred in a separatory funnel, in which water and chloroform were added at appropriate volumes, in order to achieve phase separation by adjusting the chloroform/methanol/water-based homogenate to a ratio of 1:1:0.9 (*v*/*v*/*v*). The extracted-from-breads TLs present in the lower chloroform phase were then collected in round-bottom flasks, and the solvents (mainly chloroform) were further evaporated on a flash rotary evaporator at 37 °C under vacuum (Buchi Rotavapor; Mason Technology Ltd., Dublin, Ireland). The remaining TLs were obtained from the round-bottom flasks by being re-dissolved in small volumes (1–3 mL) of a 1/1 (*v*/*v*) chloroform/methanol solution, which facilitated their transfer to small pre-weighed glass tubes. 

From these TL samples, both NL and PL extracts were obtained by counter-current distribution based on pre-equilibrated petroleum ether (for obtaining the NLs) and ethanol 87% in water (for obtaining the PLs), as previously described [7,22]. The solvents from the separated NLs and PLs were again evaporated on a flash rotary evaporator at 37 °C under vacuum, and then transferred to small pre-weighed glass tubes using small volumes (1–3 mL) of petroleum ether for NLs and a 1/1 (*v*/*v*) chloroform/methanol solution for PLs. 

The solvents used to transfer the extracted lipid samples in all tubes were evaporated under nitrogen stream, and all obtained TLs, PLs and NLs of each bread sample were then weighed to obtain the yield of extraction and then stored at −20 °C (under nitrogen) before further analysis.

### 2.3. Assessment of Anti-Platelet and Anti-Inflammatory Properties of Extracts with Light Transmitance Aggregometry

The bioassays for evaluating the anti-platelet and anti-inflammatory properties of all ACBP-FGE-PLs extracts, as well as of the TL, PL, and NL extracts from all bread samples, were performed in human platelet-rich plasma (hPRP) preparations from healthy donors (n = 6) by assessing their capacity to inhibit the aggregation of human platelets when hPRP was induced via the inflammatory and thrombotic mediator PAF and via the well-established platelet agonist ADP, in the presence of these extracts, as previously described [7,22]. The anti-inflammatory and antithrombotic potencies of each sample were expressed as means of their IC50 (half-maximal inhibitory concentrations) values ± standard deviation (SD), quantified in mass (µg) of the bioactive lipid extract present in the aggregometer cuvette that can cause 50% inhibition of hPRP aggregation induced by PAF or ADP, as previously described [7,22]. Each sample was assessed several times in blood samples obtained from different volunteers (n = 6) in order to ensure reproducibility.

### 2.4. Assessment of Fatty Acid Composition with LC–MS

Liquid Chromatography–Mass Spectrometry (LC–MS) was utilized for assessing the fatty acid composition of all ACBP-FGE-PLs extracts, as well as of the TL, PL, and NL extracts from all bread samples, as previously described [27]. Briefly, each dried extract was re-dissolved in 500 µL of dichloromethane/methanol (1:2, *v*/*v*) and then centrifuged for 6 min at 13,000 rpm (Heraeus Biofuge Stratos, Fisher Scientific Ltd., Dublin, Ireland). Filtration of the clear supernatants was performed using 3 kDa ultra-centrifuge filters (Amicon Ultra 3 k, Merck Millipore Ltd., Darmstadt, Germany). In order to obtain the fatty acid profiles in these filtrates, 10 µL of each filtrate was injected in HPLC (Agilent 1260 series, Agilent Technologies Ireland Ltd., Little Island, Co., Cork, Ireland) system equipped with Q-TOF mass spectrometer (Agilent 6520) using electrospray ionization (ESI) as the source type. The separation of fatty acids was achieved using an Agilent C18 Poroshell 120 column (2.7 µm, 3.0 × 150 mm) with gradient elution, in which the mobile phase A consisted of 2 mM ammonium acetate in water, while the mobile phase B consisted of 2 mM ammonium acetate in 95% acetonitrile. The mobile phase had initially a flow rate of 0.3 mL/min until 5 min elapsed, and increased up to 0.6 mL/min after 10 min and then held at this flow rate until the end of the run. The mass spectrometer scanned *m*/*z* from 50 to 1100, while the reference masses of 1033.988 and 112.9855 were used for monitoring the scan in negative ionization mode. The capillary voltage was 3500 V and the skimmer voltages and fragmentor were maintained at 65 V and 175 V, respectively. Drying-gas flowrate, nebulizer pressure and temperature were, respectively, set at 5 L/min, 30 psi, and 325 °C. 

The validation of the LC–MS method was performed by comparing the specific accurate mass and the retention time (RT) of several standard saturated and unsaturated fatty acids, namely, lauric (C12:0), myristic (C14:0), palmitic (C16:0), stearic (C18:0), oleic (OA, C18:1n-9 cis), linoleic (LA, C18:2n-6 cis), g-linolenic (GLA, C18:3n-6), alpha-linolenic (ALA, C18:3n-3), arachidonic (ARA, C20:4n-6), eicosapentaenoic (EPA, 20:5n-3), docosapentaenoic (DPA, 22:5n-3) and docosahexaenoic (DHA, 22:6n-3) acids (Sigma, Ireland). The standards also facilitated the assessment of each lipid extract sample, in which each of their fatty acids were identified based on their known accurate mass. The peak area of each identified fatty acid was the average of triplicate samples, and their relative content was recorded as per their average peak area. Since the peak areas do not reflect accurate amounts of individual fatty acids, the relative data must be read with caution.

### 2.5. Assessement of Total Phenolic and Carotenoid Content and Antioxidant Activities

#### 2.5.1. Total Phenolic Content (TPC) Analysis

The TPC of all ACBP-FGE-PLs extracts and the PL extracts of the novel functional breads were assessed using the Folin–Ciocalteu reagent, as previously described [28], with minor modifications. More specifically, 25 µL of each standard or extract was mixed with 125 µL of a 10-fold diluted Folin–Ciocalteu reagent in microplate wells. In order to analyze the lipophilic or hydrophilic phenolic content in the extracts, acidified ethanol (50 µL, 0.7% *v*/*v*) or sodium phosphate buffer (50 µL, 50 mM, pH 7.5) was then added. The resultant mixtures were incubated in darkness for 120 min, and the absorbance of each standard/sample assessed was measured at 755 nm. The TPC of the extracts tested were calculated by the summing up of the hydrophilic phenolic content (HPC) and lipophilic phenolic content (LPC), and the concentration was measured based on quercetin standard curve. The results were expressed as µg quercetin equivalent/g extract (DW). 

#### 2.5.2. Total Antioxidant Activity (TAA) Analysis

The colorimetric evaluation of the TAA of both the HPC and LPC of all extracts were carried out as previously described and relied on the ABTS (2,2′-azinobis-(3-ethylbenzothiazoline-6-sulfonic acid)) radical cation decolorization method, as well as the ferric-reducing antioxidant power (FRAP) method [29]. More specifically, the TAA of the all FGE-ACBP-PL extracts and the PL extracts of the novel functional breads were measured using the FRAP method [28], with some modifications. In total. 180 µL of freshly made FRAP solution was added for reaction into 20 µL of each standard or sample, and the resultant mixtures were incubated in the dark for 120 min. Then, absorbance measurements were recorded at 593 nm. The TAA values of extracts were determined by summing up the hydrophilic (HAA) and lipophilic (LAA) antioxidant activity values based on a Trolox standard curve using different concentrations. The results for the extracts were expressed in µg Trolox equivalent (TE)/g extract. 

#### 2.5.3. Total Carotenoids (TCC) Analysis

For total carotenoids content evaluation, samples of the ACBP-FGE-PLs extracts and the PL extracts of the novel functional breads were weighed and blended with cold acetone and ground with a mortar and pestle. Filtration was then performed using a Buchner glass funnel, and the residue was washed with acetone. This process was repeated until the residue was colorless. The next step involved partitioning the acetone extract to petroleum ether. A portion of the extract was combined with petroleum ether in a separatory funnel, and distilled water was added slowly without shaking to prevent emulsion formation. The two phases were allowed to separate, and the lower aqueous-acetone phase was discarded. The remaining acetone extract was transferred to petroleum ether through several repetitions, followed by washing with water to remove residual acetone. Finally, the petroleum ether phase was collected and dried with sodium sulfate [30]. Absorbance measurements were recorded at 450 nm and the results were expressed as ng β-carotene equivalent/g sample.

## 3. Results and Discussion

### 3.1. Yield Extraction of Lipids from ACBP

All ACBP-FGE contained high amounts of PLs (Table 1), as the majority of the lipids were found to be polar (>90%), which is in accordance with the literature in extracts from fruit-based by-products (apple pomace, grape pomace and citrus fruits’ by-products) that were however obtained using conventional extraction methods [5,6,7,8,9,22]. The high yield of PLs extracted with food-grade solvents from all apple pomaces further emphasizes their potential as good sources of bio-functional dietary PLs from apple pomace. 

It is noteworthy to also discuss the greater portion of PLs in the food-grade extractions, in comparison to extracts from the same samples obtained by the combination of the Bligh and Dyer and Galanos and Kapoulas extraction and separation methods [7,8,22]. More specifically, the food-grade extracted samples exhibited much greater amounts of lipids, which may be associated with the higher polarity of the food-grade solvents chosen, in comparison to the less polar conventional solvents used in the extraction of Bligh and Dyer and Kapoulas and Galanos methodology, such as chloroform and petroleum ether [22]. This increase in the ratio may indicate a more efficient extraction method as PLs were previously described as the most bioactive portion of the lipids, the NLs with very low bioactivities and the TL (PL and NL) displaying an intermediate effect [22]. The food-grade extraction utilized in the present study effectively extracted the more polar lipid and amphiphilic bioactives from apple pomaces of the three different apple varieties (Jonagold, Dabinett and Aston Bitter) with a higher yield in comparison to that observed by applying conventional methods [22] (Table 1), which are rich in bioactive PLs (more than 95% of TLs are PLs). Such ACBP-FGE-PLs extracts are bio-functional extracts rich in amphiphilic bioactives that are safe to be consumed as ingredients of food supplements and nutraceuticals, but are also safe to be infused in several food products in the food industry for human consumption.

### 3.2. Anti-Inflammatory and Anti-Platelet Properties of Food-Grade Extracts from ACBP

**Figure 1:**
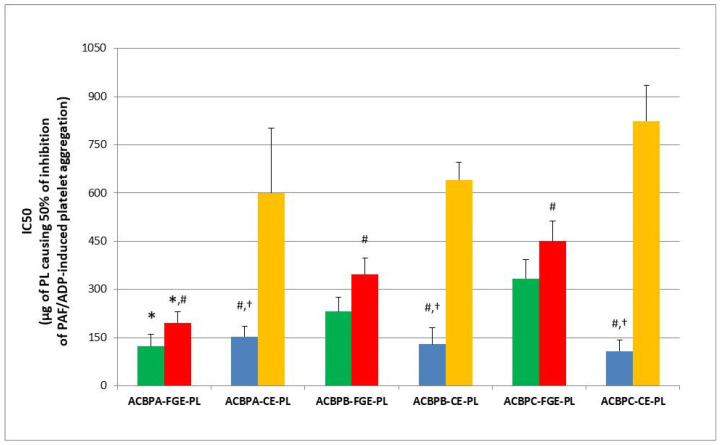
The anti-inflammatory and anti-platelet potency of food-grade extracted PLs (ACBP-FGE-PL) of apple cider by-products (ACBP) from low (ACBPA), intermediate (ACBPB) and high (ACBPC) tannin-content apple pomace, versus that of PLs extracted from these ACBP with conventional extraction methods (ACBP-CE-PL), against human platelet aggregation induced via the inflammatory and thrombotic mediator PAF (green bars depict the anti-PAF activities of ACBP-FGE-PL and blue bars those of ACBP-CE-PL) or by the platelet agonist ADP (red bars depict the anti-ADP activities of ACBP-FGE-PL and yellow bars those of ACBP-CE-PL), respectively. Results are expressed as means of the IC50 values in µg of PL in the aggregometer cuvette that causes the 50% inhibition of PAF/ADP-induced platelet aggregation (the lower the IC50 value for an extract, the higher its inhibitory effect against the specific agonist of platelet aggregation) (n = 6). * Denotes statistically significant difference (*p* < 0.05) when the anti-PAF potency (IC50 value) of the bioactive ACBP-FGE-PL of the low-in-tannins ACBPA (ACBPA-FGE-PL) were compared with that of the ACBP-FGE-PL from the other two ACBPs with higher tannin contents (ACBPB-FGE-PL and ACBPC-FGE-PL). # denotes statistically significant difference (*p* < 0.05) when the anti-PAF and the anti-ADP potencies (IC50 values) of the bioactive ACBP-FGE-PL were compared to those of the ACBP-CE-PL from the same ACBP. † denotes statistically significant difference (*p* < 0.05) when the anti-PAF potency was compared to the anti-ADP potency (IC50 values) of the bioactive ACBP-FGE-PL or the ACBP-CE-PL of the same ACBP. Results for the IC50 values against both PAF and ADP for the ACBP-CE-PL from all three types of ACBP (blue and yellow bars) are reproduced from [22] for facilitating comparisons.

The biological activities of all extracts were evaluated by acquiring their putative anti-inflammatory and anti-platelet potency against human platelet activation and aggregation induced by the inflammatory and thrombotic mediator PAF, as well as by the classic platelet agonist ADP, were assessed as previously described [7,22]. In order to facilitate comparisons between the PL bioactives of apple pomace that were extracted either by conventional methods or by food-grade methods, Figure 1 shows an overview of the anti-inflammatory and anti-platelet potency (IC50 value) of the ACBP-FGE-PLs of from apple pomace of low (ACBPA)-, intermediate (ACBPB)- and high-in-content tannins (ACBPC), respectively, against human platelet aggregation induced via the inflammatory and thrombotic mediator PAF or via a classic platelet agonist (ADP) (green and red bars depict the anti-PAF and anti-ADP activities of ACBP-FGE-PL, respectively). In the same figure, the previously reported anti-PAF and anti-ADP effects of bioactive PLs extracted from these ACBPs with conventional extraction methods (ACBP-CE-PLs) [22] are also depicted (blue and yellow bars depict the anti-PAF and anti-ADP activities of ACBP-CE-PL, respectively), in order to facilitate comparisons. The results are expressed as means of the IC50 values in µg of PLs in the aggregometer cuvette that causes the 50% inhibition of PAF/ADP-induced platelet aggregation. It should be mentioned that the lower the IC50 value for an extract, the higher its inhibitory effect against the specific agonist of platelet aggregation.

All ACBP-FGE-PLs showed strong anti-inflammatory and anti-platelet bioactivities against human platelet aggregation induced by the inflammatory and thrombotic mediator PAF and the classic platelet agonist ADP. From all apple pomaces, the apple pomace of the low-in-tannins apple variety Jonagold (ACBPA) was found to contain food-grade extracted PL bioactives (ACBPA-FGE-PLs) with the strongest anti-PAF and anti-ADP potency (lower IC-50 values), with a statistical significant difference when compared with the ACBPB-FGE-PL and ACBPC-FGE-PL from the other two intermediate- and high-in-tannins ACBPs, respectively, in which both the anti-PAF and anti-ADP effects of their ACBPB-FGE-PLs and ACBPC-FGE-PLs were of lower potencies (higher IC50 values). (*p* < 0.05 for all these comparisons). 

Thus, in contrast to the ACBP-CE-PLs, and as it is shown Figure 1, the anti-inflammatory and anti-platelet potency of the low-in-tannins ACBPA-FGE-PLs against both the PAF pathway of inflammation and thrombosis and the ADP pathway of platelet aggregation was found to be statistically significantly stronger when compared to the relative bioactivities of the higher-in-tannins ACBPB-FGE-PLs and ACBPC -FGE-PLs. These results show that more bioactive PLs are present or were able to be extracted from the low-in-tannins apple pomace ACBPA rather than from the higher-in-tannins apple pomaces ACBPB and ACBPC. For this reason, the low-in-tannins ACBPA-FGE-PLs were chosen to be further valorized by incorporating them as functional ingredients of whole-grain functional bread products.

In comparison to the cider products, the by-products, although they display lower activities against PAF and ADP in all samples, show similar and comparable results to that of apple pomace PLs extracted with conventional methods [22], while PL bioactives were extracted in higher quantities (one-order-of-magnitude-higher quantities exist in apple pomace than in apple juice and apple cider), suggesting that apple pomace is a better source for such anti-inflammatory and anti-platelet PL bioactives. 

The anti-PAF activities of each ABPC-FGE-PL were of similar potencies to their anti-ADP activities from the same ACBP (*p* > 0.05 when the anti-PAF activities of the ACBPA-FGE-PLs, ACBPB-FGE-PLs and ACBPC-FGE-PLs were compared to their relative anti-ADP effects within the same ACBP). This was not the case for the ACBP-CE-PLs in each ACBP where the anti-PAF effects were much stronger than their anti-ADP effects within the same ACBP (*p* < 0.05 in all these comparisons) [22], similarly to the conventionally extracted lipids found in apple juice [7,8]. 

Even though in each ACBP the ACBP-FGE-PLs showed stronger anti-ADP effects when compared to those of the ACBP-CE-PLs within the same ACBP (*p* < 0.05 for all these comparisons), only the ACBPA-FGE-PLs showed similar anti-PAF potency (IC50 value) to that of the ACBP-CE-PLs of the same low-in-tannins ACBPA (*p* > 0.05 for this comparison). This was not the case for the other two intermediate- and high-in-tannins ACBPB and ACBPC, respectively, in which the anti-PAF effects of their ACBPB-FGE-PLs and ACBPC-FGE-PLs were of lower potency (higher IC50 values) compared to the ACBP-CE-PL for these two ACBPs (*p* < 0.05). Based on the above, the ACBPA-FGE-PLs from the low-in-tannins-content apple cider by-product (ACBPA) seems to be the most promising ACBP-FGE-PL to be used either for producing novel food supplements with strong anti-inflammatory and anti-platelet potency and promising cardio-protective properties and preventative potential against inflammation-related chronic disorders or for the fortification of other foods in order to produce a novel functional food product with strong anti-inflammatory and anti-platelet potency and promising cardio-protective properties too. For this reason, several concentrations of the ACBPA-FGE-PLs were utilized for the fortification and production of novel whole-grain breads with functional anti-inflammatory and anti-platelet potency and promising cardio-protective properties.

### 3.3. Fatty Acid Composition of Food-Grade Extracted Bioactive PLs from ACBP

The fatty acid composition of the ACBP-FGE-PLs extracts from all ACBP apple pomaces was elucidated through LC–MS analysis, and the results are displayed in Table 2. All PL extracts were found to be rich in polyunsaturated fatty acids (PUFAs), followed by lower amounts of saturated fatty acids (SFA) and the less abundant monounsaturated fatty acids (MUFA). More specifically, all bioactive ACBP-derived PL extracts contained high amounts of the most abundant essential omega-6 (n-6) PUFAs, linoleic acid (LA) (C18:2 c9, 12 n-6), followed by the essential omega-3 (n-3) PUFAs, alpha-linolenic acid (ALA) (C18:3 c9,12,15 n-3).

The results also display a variety of fatty acids that are similar to those previously reported from PL-extracts from the same orchard and breed; however, the composition of each sample is different. For example, the n-6 PUFA LA was found to be more abundant and the n-3 PUFA ALA much less abundant in the food-grade extracts, and subsequently the n-6/n-3 PUFA ratios of the food-grade extracted PL bioactives were much higher in comparison to the relative levels for these PUFA and n-6/n-3 PUFA ratios previously reported in conventionally extracted PL-bioactives from these three apple pomaces [22]. In addition, long-chain n-3 PUFA like EPA, DPA and DHA were not detected in the food-grade PL extracts from any of the apple pomaces assessed in comparison to the very low but considerable amounts of these LC n-3 PUFA that were previously detected in conventionally extracted PL bioactives from these three apple pomaces [22]. 

These alterations in the fatty acid composition and structure activity relationships between the food-grade extracted and the conventionally extracted PL-bioactives seem also to be associated with the lower efficacy of the food-grade extraction method to extract PLs containing more hydrophobic fatty acids with longer chains like the n-3 LC-PUFA (EPA, DPA and DHA), in comparison to the more hydrophobic organic solvents (chloroform and petroleum ether) used in the conventional extraction methodology, in which low but considerable amounts of such PL containing n-3 LC-PUFA like EPA, DPA and DHA were detected [22]. Since such long-chain PUFAs are usually trapped within the pectin of apple pomace [31], it seems that the milder and more polar food-grade solvents cannot break these complexes to extract these long-chain PUFA bioactives, in contrast to the more intense chloroform-based non-food-grade conventional extraction methods. These differences were expected with the change in lipid extraction procedures as it has been recorded in the literature that different extraction methods can produce different fatty acid compositions [32]. 

Nevertheless, this result did not affect the bioactivities of the FGE-ACBP-PL, suggesting that other fatty acids bound in the food-grade extracted PL seems to participate in their anti-inflammatory properties like the ALA and bioactive monounsaturated fatty acids (MUFA) like oleic acid (OA, C18:1c9), which was the most abundant MUFA in all PL extracts of the three ACBPs. The MUFAs, however, were found to be in considerably lower amounts than the SFA and PUFA contents of all PLs from ACBPs, with exadecenoic acid (C16:0) being the most abundant SFA.

These results are in accordance with the previously reported fatty acid content of the bioactive PL extracts of apple products (apple juice and cider) from the same apple varieties (Jonagold, Dabinett, Aston Bitter) and of the PC bioactives of the PL extract of apple juices from the low-in-tannins Jonagold apple variety [7,8], and in apples and apple pomaces in general [33,34], while they again further suggest that such PL bioactives rich in n-3 PUFAs seem to migrate equally to their apple juice/cider products and the relevant ACBP apple pomace remnants/wastes during processing. Interestingly, the PUFA composition of the ACBP-FGE-PLs had comparable n-3 PUFA content and n-6/n-3 PUFA ratio with those observed in conventionally extracted lipids from the apple pomaces of the Jonathan and Golden Delicious apple varieties, but a much higher content of n-3 PUFA and thus a much lower n-6/n-3 PUFA ratio than those observed in conventionally extracted lipids from the apple skin of these two varieties, Jonathan skin and Golden Delicious skin, respectively [34].

In addition, the presence of such essential an n-3 PUFA like ALA bound in the bioactive PL extracts of the ACBP further supports their anti-inflammatory potency and provides a rationale for their strong anti-PAF effects. Such dietary PLs rich in n-3 PUFA from fruits’ by-products have been found to inhibit platelet aggregation induced via the inflammatory and thrombotic mediators PAF and thrombin, but also via classic well-established platelet agonists such as collagen and ADP [5,6,9,22], as was also observed in the present study for the rich in n-3 PUFA bioactive ACBP-FGE-PLs. 

Nevertheless, apart from the bioactivities observed on the PL bioactives, the n-3 PUFA content of these PLs has on its own several beneficial bio-functionalities, especially when released from these PLs in cells via specific cytoplasmic phospholipases’ A2 (PLA_2_) enzymatic activities [5]. For example, the PLA_2_-based release of n-3 PUFA from such bioactive PLs in cell membranes and/or lipoproteins facilitates the production of anti-inflammatory eicosanoids that act antagonistically to other inflammatory and thrombotic eicosanoids (prostaglandins, leukotrienes, and thromboxanes) usually produced via n-6 PUFAs like arachidonic acid [5]. The latter further supports the health benefits derived from the aforementioned n-3 PUFA ALA, while healthy dietary patterns based on these n-3 PUFA have shown strong preventative benefits against several chronic disorders, such as in a Mediterranean diet enriched in ALA for the secondary prevention of coronary heart disease [35,36,37,38]. 

In the ACBP-FGE-PLs, each sample displayed considerable levels of the ALA n3PUFA and of the OA MUFA that can be considered beneficial against thrombo-inflammatory mediators and associated diseases [35,36,37,38,39]. More specifically, such PL bioactives from fruits’ by-products that are rich in the n3 PUFA ALA and the MUFA OA have been associated with anti-PAF and anti-ADP properties [5,6,9,22]. 

The differences observed between the fatty acid content of the ACBP-FGE-PL with that of the previously assessed fatty acid content of the ACBP-CE-PLs [22], seem to also be associated with the lower anti-inflammatory and anti-platelet potency observed in the intermediate-in-tannins ACBPB-FGE-PL and the high-in-tannins ACBPC-FGE-PL against PAF in comparison to the higher bioactivities observed in the relevant ACBP-CE-PL bioactives for these two apple pomaces. In contrast, in these apple pomaces, the food-grade extracted PL bioactives exhibited higher anti-ADP activities than those of their conventionally extracted PL-bioactives. 

In contrast, such differences were not observed in the low-in-tannins ACBPA, in which the ACBPA-FGE-PLs showed similar anti-inflammatory potency against PAF and much stronger anti-platelet efficacy against ADP in comparison to the ACBPA-CE-PL bioactives. These results further suggest that it is not only the fatty acid content that provides bio-functionality in the food-grade extracted PL bioactives from apple pomace, rather than their overall structures, while it seems that the more polar food-grade extraction methods can extract more polar lipid bioactives, such as glycolipids and amphiphilic phenolic compounds, which further contribute to the overall anti-inflammatory potency for these PL-extracts against both the inflammatory and thrombotic pathways of PAF, as well as against the platelet activation pathways of ADP. Nevertheless, more targeted studies on structural elucidation are needed in order to fully evaluate the structure-activity relationships of the food-grade extracted apple pomace PL-bioactives, in comparison to the conventionally extracted PL.

### 3.4. Yield of Lipid Extraction from Novel Functional Breads Infused with Apple Pomace Lipids

The total lipids (TLs) from all bread samples were extracted using the Bligh and Dyer extraction method previously described by Tsoupras and others [7,22]. The TLs of these samples were further separated into polar and neutral lipids using the Kapoulas and Galanos counter-current distribution method, also described by Tsoupras and others [7,22]. 

Table 3 below displays the results of these extractions and separations (expressed as g of lipids/100 g sample and also as % percentages of PLs or NLs within the TL content of the breads).

Interestingly, the bread samples displayed a slight decrease in TL content with the addition of apple pomace lipid extract with the exception of medium concentration. This also came with an increase in the polar lipid percentage and a subsequent decrease in the neutral lipid percentage. A high concentration of apple pomace lipid-infused bread (HB) displayed the lowest amount of total lipids with the greatest amount of polar lipid content in comparison to the other bread samples. This may suggest that the increase in the added lipid causes an overall decrease in total lipid content with an increase in the desirable polar portions of lipids. This was found in some bakery products previously recorded by Wang, H.J. and Thomas, R.L. [40], where the direct addition of apple pomace displayed a lowering of the fat content. There is little research into the polar and neutral lipid composition of breads that have been infused with apple pomace lipids. Future research is required to determine the effects on the lipid yield of breads with the addition of apple pomace extract. Although these altercations are considerably low, it should be noted that the added apple pomace lipids were in very low quantities in comparison to the breads themselves, in order to provide any substantial differences in the yield of extracted lipids from these breads. 

### 3.5. Anti-Inflammatory and Anti-Platelet Potency of Lipid Bioactives from the Novel Functional Whole-Grain Breads Infused with Several Concentrations of the Low-in-Tannins ACBPA-FGE-PLs Bioactives

**Figure 2:**
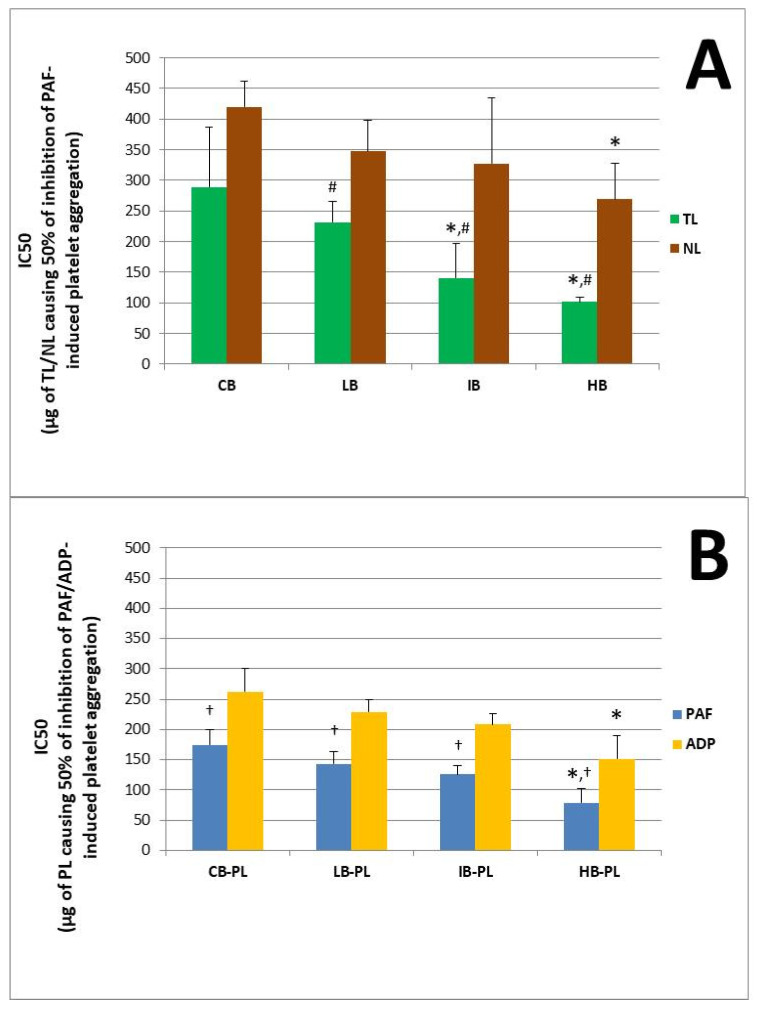
The anti-inflammatory and anti-platelet potency of lipid bioactives from the novel functional whole-grain breads infused with several concentrations of the low-in-tannins ACBPA-FGE-PLs bioactives against human platelet aggregation induced via the inflammatory and thrombotic mediator PAF or by a classic platelet agonist, ADP. (**A**) depicts the anti-PAF potency (IC50 values) of the total lipids (TL; green bars) and neutral lipids (NL; brown bars) extracted and separated from the novel functional whole-grain breads containing low (LB), intermediate (IB) and high (HB) contents (concentrations) of the infused ACBPA-FGE-PL bioactives, versus control whole-grain bread (CB) that was not infused with ACBPA-FGE-PL bioactives. (**B**) depicts the anti-PAF (blue bars) and anti-ADP (yellow bars) potency (IC50 values) of the bio-functional polar lipids (PL) extracted and separated from novel functional whole-grain LB, IB and HB breads that were infused with ACBPA-FGE-PL bioactives, versus control whole-grain CB bread. Results are expressed as means of the IC50 (half-maximal inhibitory concentrations) values in µg of bread lipid extract in the aggregometer cuvette that causes the 50% inhibition of PAF/ADP-induced platelet aggregation (the lower the IC50 value for a lipid extract the higher its inhibitory effect against the specific agonist of platelet aggregation). * denotes statistically significant difference (*p* < 0.05) when the anti-PAF or anti-ADP potency (IC50 value) of the bioactive TLs and NLs (**A**) or PLs (**B**) of the HB and IB breads are compared with those of the other two breads (CB and LB). # denotes statistically significant difference (*p* < 0.05) when the anti-PAF effects of the TL bioactives were compared with those of the NL within the same bread samples. † denotes statistically significant difference (*p* < 0.05) when the anti-PAF potency of the PL bioactives was compared to their anti-ADP potency (IC50 values) within the same bread sample. (n = 6).

The anti-inflammatory and anti-platelet potency (IC50 value) of lipid bioactives from the novel functional whole-grain breads infused with several concentrations of the low-in-tannins ACBPA-FGE-PLs bioactives against human platelet aggregation induced via the inflammatory and thrombotic mediator PAF or via a classic platelet agonist (ADP) are depicted in Figure 2A,B. More specifically, Figure 2A depicts the anti-PAF potency (IC50 values) of the TL (green bars) and NL (brown bars) extracted and separated from novel functional whole-grain breads containing low (LB), intermediate (IB) and high (HB) contents (concentrations) of the infused ACBPA-FGE-PL bioactives, versus the control whole-grain bread (CB) that was not infused with ACBPA-FGE-PL bioactives. In addition, Figure 2B depicts the anti-PAF (blue bars) and anti-ADP (yellow bars) potency (IC50 values) of the bio-functional PLs of the novel functional whole-grain LB, IB and HB breads, versus the CB control whole-grain bread. Again, results are expressed as means of the IC50 values in µg of bread-derived PLs in the aggregometer cuvette that causes the 50% inhibition of PAF/ADP-induced platelet aggregation. It should again be stressed out that the lower the IC50 value for a bread-derived PL extract, the higher its inhibitory effect against the specific agonist of platelet aggregation.

In the present study, it was found for the first time that in all breads assessed, their PLs were the most bioactive lipid extracts against PAF, followed by TL that had intermediate anti-PAF potency, and then NLs that showed the lowest bioactivities. 

The TLs of all LB, IB and HB bread samples had statistically significantly higher anti-PAF potencies when compared to the relevant anti-PAF effects of the NL extracts of these breads (*p* < 0.05 for these comparisons). Thus, in these breads, their TLs had an anti-PAF potency with IC50 values closer to those of their PL bioactives. This was not the case for the TLs of the CB, in which the very low anti-PAF effect was of similar low potency to that of their NL extracts (*p* > 0.05 for these comparisons).

PL bioactives from the HB showed the highest anti-PAF potency (lowest IC50 values) in comparison to the bioactivities of the PL form all the other breads against the PAF-pathway (*p* < 0.05 for all these comparisons). Also, the strong anti-inflammatory potency of the PL-bioactives extracted and separated from the novel functional HB bread is comparable to the relative ones previously observed in other fruit-derived functional foods, beverages and by-products [5,6,7,8,9,22,27].

In addition, the TLs from both the HB and IB showed also statistically significantly higher anti-PAF activities (lower IC50 values) when compared to the TLs from the LB and CB (again, *p* < 0.05 for these comparisons). This further suggests that the higher the addition of the apple pomace lipids, the stronger the bioactivities observed in the infused breads against the inflammatory pathways of PAF-induced platelet aggregation. 

Since the PLs were the most bioactive lipids of all infused breads against PAF, they were also chosen to be tested against ADP too, in a similar way to previous research for apple products and by-products [7,8,22]. By this assessment, it was also found that the PL bioactives from the HB again exhibited the highest anti-ADP potency (lowest IC50 values) in comparison to the bioactivities of the PL from all the other breads against the ADP pathway (*p* < 0.05 for these comparisons).

It should be noted that the anti-PAF activities of the PL bioactives in each bread were statistically significantly stronger (lower IC 50 values) when compared to their relative anti-ADP activities within the same bread sample (*p* < 0.05 for these comparisons), which indicates that the bread PL bioactives have higher specificity against the inflammatory and thrombotic PAF pathway rather than against the ADP pathway of platelet aggregation. Nevertheless, both bioactivities were within the same order of magnitude against both ADP- and PAF-associated pathways for the PL bioactives in each bread sample, indicating the strong cardio-protective potential for these functional breads against two distinct thrombo-inflammatory pathways.

Overall, the above observed findings further suggest that the infusion of apple pomace bioactive PL into whole-grain bread produced a novel functional bread (HB) with strong anti-inflammatory and anti-thrombotic potency against the PAF pathway and anti-platelet bioactivities against the ADP pathway of human platelet aggregation. Subsequently, such novel bio-functional breads that were developed in the present study by utilizing ACBP-FGE-PL bioactives as functional ingredients, with strong anti-inflammatory and anti-platelet properties, seem to provide more enhanced cardio-protection and preventative properties against inflammation-related chronic disorders than the classic whole-grain breads (CB) that do not contain these functional ingredients. Nevertheless, more studies are needed, mainly in vivo dietary interventions, in order to fully elucidate such an anti-platelet and anti-inflammatory potential for the novel bio-functional breads produced in the present study.

### 3.6. Fatty Acid Profile of the PL from Bread Infused with Apple Pomace PL

Table 4 displays the fatty acid profile of the PL extracts from all novel functional whole-grain LB, IB and HB breads that were infused with ACBPA-FGE-PL bioactives, versus the fatty acid content of the PL extracts from the control whole-grain CB bread. These samples showed many similarities in their fatty acid content; however, the addition of ACBP-FGE-PL extract caused some differences in their composition in specific fatty acids, as expected. Such differences in samples can be attributed solely to the addition of lipids, as there have been no other changes to the sample’s constituents/ingredients and the baking process. These results come in accordance with previously analyzed bread samples that were also enriched with functional lipids and fatty acids [41]. 

The PL extracts from these breads displayed an abundance of unsaturated fatty acids (UFA) (>78%) with the remaining 18–22% being composed of SFA. The most abundant fatty acids in all the breads were the PUFAs with a range of 50–55%, followed by MUFA which ranged from 25–28%. Within the high levels of PUFA, the most abundant was again the omega 6 LA PUFA followed by the omega 3 ALA PUFA in all samples. Notably, the levels of the n6/n3 PUFA ratio for the PLs from all these bread samples were found to be approximately 4.5–6.5 and thus within the favorable range of healthy foods/diets and much lower than the unfavorable reported ones (>15) for western-type processed foods and diets [35]. It has been reported that the lower the value for this ratio in a food, the better the health outcomes against inflammation-related chronic disorders [35]. 

Nevertheless, these favorable low values of the n6/n3 PUFA ratio were also observed in the control CB bread too, suggesting that the added amounts of the ACBPA-FGE-PLs did not affect this ratio in the PLs of all bread samples. It seems that the same baking process applied to all breads affects similarly their PUFA content, which further suggests that higher amounts of ACBPA-GE-PLs may be needed to be infused in the bread in order to modulate the breads’ PUFA content independently of the baking process. 

With respect to the MUFA content of these samples, again the n9 OA was the most abundant, followed by small but considerable portions (>4%) of palmitoleic acid (16:1). Interestingly, the content of palmitic acid was increased in the PL samples of all the breads that were infused with the ACBPA-FGE-PL, in comparison to the CB, which may further imply a saturation effect of baking on the UFA content of PL. The SFA displayed the lowest percentage in comparison to MUFA and PUFA. However, this group consisted primarily of palmitic acid (91–92% of SFA). 

Although there were small changes to the FA profiles of the breads that were infused with the lipids, it must be noted that there was an addition of 1% net weight added to the higher concentrated bread. The changes observed in the fatty acid composition can be attributed to a very low amount of added lipids. These lipids are also highly sensitive to oxidation which may have occurred during the proving and baking process due to the heat treatment and access to the air surrounding the bread. By such an oxidation of the long chain UFA, it is possible that the MUFA with a lower chain may be derived, such as the palmitoleic acid and/or an SFA like the palmitic acid, which may explain their increased levels in these breads.

Such few differences in the fatty acid composition in the PL extracts of these breads seem not to affect the bioactivities against platelet aggregation mediators (PAF and ADP). For example, the HB bread’s fatty acid profile seems to be quite similar in PUFA, MUFA and SFA content to that of the CB breads. However, the anti-inflammatory properties of the HP-derived PLs are at least twice stronger than the PL of the CB. This was the case for their activity against the ADP pathway too. These outcomes further indicate that other parameters in all the breads assessed are responsible for the improvement of their functional anti-inflammatory and anti-platelet cardio-protective properties, rather than just the fatty acid profile of their PLs. 

Nevertheless, it should be stressed that if bioactive PLs are present in foods, beverages and/or food supplements, after their consumption, some amounts (5–20%) of these dietary bioactive PLs (with n-3 PUFA or MUFA at their sn-2 position) are not usually digested and due to their amphiphilic properties they are absorbed and diffused from the intestine to the blood stream where they preferably are incorporated into plasma lipoproteins and, from there, to several blood cells and tissues, including platelets, but also to tissues with accessibility issues, such as the brain [1,5]. Their amphiphilic nature facilitates this journey within the blood stream and their incorporation into lipoproteins, cell membranes and for surpassing difficult-to-access barriers like the blood–brain barrier. The incorporation of such PL bioactives in lipoproteins like LDL and HDL cholesterol reduces beneficially their oxidative and inflammation-inducing status, while they beneficially increase the levels of the “good” HDL cholesterol, as well as also inducing the anti-oxidant and anti-inflammatory enzymatic activities of HDL with anti-inflammatory health benefits [1,5,42]. 

Moreover, the binding of such PL bioactives to the membranes of blood cells like leukocytes and platelets, but also to the membranes of endothelial cells, directly or indirectly, inhibits several thrombo-inflammatory cascades [1,2,5]. For example, PL bioactives from several foods have been found to reduce PAF synthesis in several cells, and thus reduce PAF levels in blood with subsequent beneficial reduction in the inflammatory status of blood and blood vessels [1,2,5]. In addition, such food-derived PL-bioactives interact directly through a strong inhibitory antagonistic or a weak agonistic effect or both effects (in different concentrations) against the PAF and thrombin pathways of activating cells, including platelets, reducing thus the activation of these cells, including the inhibition of platelet aggregation, because of their structural resemblance to the PAF molecule and thus due to antagonism against the binding of PAF on its receptor [1,2,5].

Some of these PL bioactives can also inhibit beneficially platelet aggregation indirectly due to their bio-functional fatty acid composition. More specifically, when bound to the membranes of platelets, these phospholipids are substrates for the enzyme activity of PLA_2_ with a subsequent release of their bioactive MUFAs and n-3 PUFAs from the *sn*-2 position of their structures into the cytoplasm, whereby in binding to specific proteins they are able to affect several thrombotic and inflammatory intracellular signaling pathways and gene expression [1,2,5]. 

For example, the released UFAs from such PL bioactives can additionally affect the formation of pro-inflammatory eicosanoids and thus inhibit their involvement in platelet aggregation [1,5,35,36,37,38,39]. UFA released intracellularly from dietary PL can inhibit specific inflammatory cyclooxygenases (COX) that are the basic enzymes involved in synthesis of the pro-inflammatory eicosanoids, which are usually induced as an after-effect of the PAF- or thrombin-induced release of arachidonic acid. Subsequently, the indirect inhibition of the PAF-/thrombin-induced intracellular cascades for the aggregation of platelets can also be associated with the UFA content of dietary PL bioactives [1,5].

Several other mechanisms have also been proposed for the direct inhibitory effects of UFA like the OA and ALA against platelet aggregation [35,36,37,38,39], suggesting that the release of such bio-functional UFA of the PL bioactives in the ACBP and in the novel bread products may be able also to contribute in a reduction in the risk of platelet activation and aggregation induced by several pathways and mediators, including the PAF and ADP pathways, with several anti-inflammatory and antithrombotic health benefits, especially against the risk for inflammation-related chronic disorders. 

### 3.7. The Total Phenolic and Carotenoid Content and Antioxidant Activities of the Food-Grade Extracted Polar Lipids from the Apple Cider By-Products (Apple Pomace), as Well as of the Polar Lipids from the Novel Functional Breads Containing the Apple Pomace Extracts versus Control Breads

The total phenolic and carotenoid content of the ACBP-FGE-PL, as well as those of the PLs from all the novel functional breads infused with the ACBPA-FGE-PL versus the CB breads are shown in Table 5. 

From all ACBP-FGE-PL extracts assessed, the apple pomace from the intermediate-in-tannins apple variety (ACBPB) showed the highest hydrophilic, lipophilic and total phenolic content, as well as total carotenoid content, followed by that of the apple pomace from the high-in-tannins apple variety (ACBPC), with the apple pomace from low-in-tannins apple variety (ACBPA) showing the lowest hydrophilic, lipophilic and total phenolic content, as well as total carotenoid content. Notably, the total phenolic content of all ACBP-FGE-PLs were comparable to those previously observed in four other apple varieties (Rome Beauty, Idared, Cortland and Golden Delicious), in which the peel contained many more phenolics than the flesh [43]. 

Moreover, the carotenoid content of the ACBPB-FGE-PL and ACBPC-FGE-PL extracts showed comparable carotenoid contents with those of other apple varieties [44], while the ACBPA-FGE-PLs showed much lower carotenoid contents, which may explain why no carotenoids were detected in the PLs of all bread samples infused with this extract. This, however, was also observed in the control CB breads too, which were not infused with the ACBPA-FGE-PLs extract, suggesting that the carotenoids’ content in all breads was independent to the presence or not of the apple pomace bioactives, rather than the baking process that seems to have saturated all carotenoids.

Interestingly, in all the produced novel functional LB, IB and HB breads that were infused with several concentrations of the food-grade extracted polar lipids from this apple pomace (ACBPA-FGE-PL), the hydrophilic phenolic content was statistically higher than that of the control CB breads that did not contain any of the functional extracts from apple pomace. In all breads, no carotenoid content was detected. 

The antioxidant activity of the ACBP-FGE-PL, as well as those of the PL from all the novel functional breads infused with the ACBBPA-FGE-PL versus the CB breads, are shown in Table 6. For evaluating the anti-oxidant activity of these samples, the FRAP assay (ferric-reducing antioxidant power assay) and the ABTS assay, compared with a Trolox (water-soluble vitamin E analogue) standard, were applied as previously described [28,29]. The ABTS assay measures the free radical scavenging activity, meaning the relative ability of antioxidants to scavenge the ABTS generated in aqueous phase, while the FRAP assay measures the antioxidant potential in samples through the reduction of ferric iron (Fe^3+^) to ferrous iron (Fe^2+^) by antioxidants present in the samples, meaning a test measuring the ferric-reducing ability of plasma [28,29,45]. 

The differences in the substrate used (an organic radical producer was used in one method, while the other method works with a metal ion for oxidation), as well as the differences in the reaction procedures in these two experimental approaches, seem to explain the big differences observed in the outcomes derived in each assay for the same sample, as shown in Table 5. Nevertheless, despite these differences, the results of these in vitro assays give an idea of the protective efficacy of the apple pomace-derived supplement and the novel functional bread products, and we applied two different experimental procedures as it is strongly recommended to use at least two methods due to the differences between the test systems investigated [46]

Interestingly, all the food-grade extracted PLs from each apple pomace showed strong hydrophilic, lipophilic and total antioxidant activities in both FRAP and ABTS assays, which were comparable to that of a standard phenolic compound, quercetin, with a known and well-established anti-oxidant capacity and subsequent health benefits. Thus, these results show that the food-grade PL extracts of apple pomace have strong anti-oxidant potency, which further supports their use for producing novel functional foods and/or food supplements/nutraceuticals with anti-oxidant cardio-protective health benefits too.

Most importantly, in all the produced novel functional breads that were infused with several concentrations of the food-grade extracted polar lipids from the apple pomace of the low-in-tannins apple variety (ACBPA-FGE-PL), the hydrophilic, lipophilic and total antioxidant activities in the FRAP were statistically significantly higher than that of the control breads that do not contain any of the functional extracts from apple pomace. This result indicates that the infusion of apple pomace food-grade extracted PL (ACBPA-FGE-PL) into the ingredients for making breads produced novel functional breads with strong anti-oxidant properties.

Within the present study, the co-extraction of phenolics into the apple pomace PL extract was achieved, which were also infused within the functional breads produced. The co-presence of phenolics in these ACBP-PL extracts provides not only anti-oxidant protection for their PL bioactives, but also several health benefits against oxidative stress and blood plasma oxidation with several health-promoting effects [1,2,5,14,19]. Moreover, the infusion of whole-grain breads with apple pomace extracts rich in PLs and phenolics resulted in an increase in their phenolic content and antioxidant capacity too, in contrast to the control breads. 

Taking into account that apart from the antioxidant benefits observed, several phenolics also possess strong anti-inflammatory potency against platelet aggregation and the activities of PAF, but also have an inhibitory effect against PAF-synthesis, which further reduces the levels of this inflammatory mediator and thus the inflammatory status and PAF associated manifestations, but also several other inflammatory pathways and related disorders [1,2,5,47], further support the strong anti-inflammatory health promoting potential for both the apple pomace extracts and the functional products, in which these extracts are infused, such as in the functional breads assessed in this study. Nevertheless, more tests are needed to fully evaluate the association of the health-promoting properties of these food-grade extracts of apple pomace and their functional food products with their amphiphilic phenolics that are co-extracted with PLs in these conditions applied, as well as on the synergism(s) of these two important molecular classes of apple pomace bioactive ingredients.

## 4. Conclusions

Within the present study, co-extraction with food-grade techniques was successful in producing extracts enriched with bioactive PLs, as well as of other functional compounds including phenolics and carotenoids. This was achieved for the first time using bio-wastes/by-products obtained during the processing of apple-related products (apple juice and cider), such as the ACBP apple pomace. These ACBP-derived extracts enriched in PLs and phenolic bioactives were found to possess strong anti-oxidant and anti-inflammatory properties, mainly against oxidative stress and especially against the inflammatory and thrombotic mediator PAF, but also had considerable anti-platelet benefits against other well-established classic platelet agonists, such as ADP. 

In addition, almost one order of magnitude higher yield of the bioactive PLs and phenolics extracts was achieved with the food-grade solvents and extraction methodology, compared to the previously reported yield for bioactive PLs from apple juice and cider, suggesting that apple pomace is a good sustainable source for such bioactive PLs and phenolics with anti-oxidant, anti-inflammatory and anti-platelet potency. Moreover, the food-grade extracts of apple pomace displayed considerably higher content in PL bioactives than those of the conventional extraction procedures, with considerable differences in the fatty acid profile. Furthermore, the food-grade extracted PL-bioactives from apple pomace showed strong anti-inflammatory and anti-platelet properties against both PAF and ADP pathways of thrombo-inflammation. 

The novel apple pomace extract, which was produced with more polar solvents than those applied in conventional extractions, was found to contain both phenolics and PL bioactives, and this facilitated the co- infusion of such dietary bioactives into the ingredients for making bread, which resulted in the successful production of novel functional breads with higher PL and phenolic bio-functionalities with both stronger anti-oxidant protection and anti-inflammatory and anti-platelet properties, with potential health benefits, in contrast to control breads that were not infused with such apple pomace bioactives. More specifically, the bioactive ACBPA-FGE-PLs derived from the lowin-tannins ACBPA showed a stronger anti-PAF and anti-ADP potency, and thus it was chosen as a functional ingredient for producing novel functional food products like whole-grain breads. The addition of 0.5–1.0 g of food-grade extracted PL bioactives from apple pomace in the ingredients for bread-making showed that novel bio-functional bread products can be developed with stronger anti-oxidant, anti-inflammatory and anti-platelet potency than the standard control breads, independently of the slight alterations to their fatty acid profile observed. These differences were unveiled by the LC–MS analysis, since the PLs from all ACBPs and the produced functional breads were shown to possess a significantly enriched UFA profile, as they were rich in both OA and the essential LA and ALA, which further support their anti-inflammatory potential. 

These outcomes also emphasize the potential valorization of apple pomaces as sustainable sources of phytonutrients, including UFA-enriched PL bioactives with strong anti-oxidant, anti-inflammatory and anti-platelet potential. Furthermore, such valorized AP-derived phytonutrients are useful for producing functional foods like whole-grain breads, with demonstrated enhanced nutritional and functional ingredients displaying beneficial health-promoting properties against oxidative stress, inflammation and associated disorders.

However, more tests are needed to further evaluate the in vivo dose-efficacy of the antioxidant and anti-inflammatory properties. This also includes the safety of the antithrombotic effects in terms of any adverse effects (e.g., for patients on blood thinners) through accumulation in food of either the bioactive extract or the produced functional food product. Sensory analysis to assess the organoleptic properties of these novel functional foods is also important, in which consumers’ acceptance of the infused product will also be assessed prior to their release in the market.

## 5. Patents

Tsoupras A, Moran D. “Bioprospecting of food grade anti-Inflammatory, anti-oxidant and Cardio-protective ingredients in APPLE by-products for added value novel functional food products and nutraceuticals (BIC-Apple)”. Invention Disclosure: IDF 2006504, Technology Transfer Office of the University of Limerick, 2021, Ireland.

## Figures and Tables

**Table 1:** Yield of food-grade (FGE) extracted PL extracts from apple cider by-products ACBPA, ACBPB, and ACBPC, expressed as g/100 g ^1^.

Samples	ACBP-FGE-PLs	ACBP-CE-PLs ^2^
ACBPAs	1.59 ± 0.23	0.25 ± 0.12
ACBPBs	1.66 ± 0.11	0.42 ± 0.21
ACBPCs	2.24 ± 0.15	0.09 ± 0.14

^1^ expressed as mean ± SD (n = 3); ^2^ Reproduced from Tsoupras et al. [22] for facilitating comparisons. Abbreviations: ACBP-FGE-PLs, food grade extracted polar lipid extracts from apple cider by-products; ACBP-CE-PL, conventional extracted polar lipid extracts from apple cider by-products; ACBP, apple cider by-product; ACBPA apple cider by-product from the low-in-tannins apples, ACBPB apple cider by-product from the intermediate-in-tannins apples, ACBPA apple cider by-product from the high-in-tannins apples.

**Table 2:** The fatty acid profile of the food-grade extracted PL bioactives for each apple pomace type, expressed for each FA as the mean value percent composition of the total fatty acids in each sample assessed (mean ± standard deviation (SD); n = 3).

Fatty Acid Name	ACBPA-FGE-PLs	ACBPB-FGE-PLs	ACBPC-FGE-PLs
C8:0	ND	ND	0.0009 ± 0.001
C9:0	0.08 ± 0.02	0.09 ± 0.03	0.06 ± 0.01
C10:0	ND	0.01 ± 0.007	0.01 ± 0.004
C11:0	0.03 ± 0.01	0.04 ± 0.03	ND
C12:0	0.03 ± 0.01	0.09 ± 0.03	0.02 ± 0.002
C13:0	ND	0.06 ± 0.01	ND
C14:0	0.59 ± 0.09	0.2 ± 0.12	0.55 ± 0.11
C15:0	0.07 ± 0.03	0.19 ± 0.06	0.06 ± 0.01
C16:0	32.96 ± 0.91	33.98 ± 1.77	25.19 ± 0.76
C16:1	0.44 ± 0.06	1.03 ± 0.26	1.17 ± 0.09
C17:0	0.24 ± 0.09	ND	0.32 ± 0.11
C18:0	7.47 ± 0.53	14.60 ± 2.17	5.08 ± 0.64
C18:1 c9	24.18 ± 0.83	22.83 ± 1.33	29.16 ± 0.46
C18:2 c9,12	29.79 ± 1.25	24.58 ± 0.1	34.59 ± 0.69
C18:3 c9,12,15	1.31 ± 0.09	0.7 ± 0.1	2.58 ± 0.01
C18:4 C9,12,15,18	ND	ND	0.01 ± 0.01
C19:0	ND	ND	0.01 ± 0.007
C20:0	0.56 ± 0.09	ND	0.22 ± 0.03
C20:1 C11	ND	ND	0.07 ± 0.07
C20:2 c11,14	0.32 ± 0.02	0.06 ± 0.01	0.5 ± 0.04
C20:3	ND	ND	0.04 ± 0.006
C20:4	ND	ND	0.006 ± 0.007
C20:5	ND	ND	0.36 ± 0.03
C24:0	1.93 ± 1.04	1.54 ± 0.78	ND
SFA	43.93 ± 2.8	50.69 ± 5.01	31.53 ± 1.71
MUFA	24.65 ± 0.91	23.90 ± 1.64	30.39 ± 0.63
PUFA	31.42 ± 1.38	25.34 ± 0.22	38.08 ± 0.79
n6	30.11 ± 1.28	24.64 ± 0.12	35.14 ± 0.74
n3	1.31 ± 0.1	0.70 ± 0.10	2.95 ± 0.05
n6/n3	23.14 ± 1.11	35.92 ± 5.43	11.93 ± 0.26

Abbreviations: FGE-PLs, food-grade extracted polar lipids, ACBPA, apple cider by-products of low-in-tannins Jonagold apple variety; ACBPB, apple cider by-products of medium-in-tannins Dabinett apple variety; ACBPC, apple cider by-products of high-in-tannins Aston Bitter apple variety; n-3, omega-3 PUFA; n-6, omega-6 PUFA; PUFAs, polyunsaturated fatty acids; MUFAs, monounsaturated fatty acids; SFAs, saturated fatty acids; ALA, alpha linolenic acid; LA, linoleic acid; EPA, eicosapentaenoic acid; DPA, docosapentaenoic acid; DHA, docosahexaenoic acid; ND, non-detectable. ND is defined as fatty acids detected with lower than 0.005% contribution to the overall fatty acid content.

**Table 3:** The yield after extraction of the total lipids (TLs), neutral lipids (NLs) and polar lipids (PLs) expressed as % of apple pomace and apple pomace lipid-infused breads (mean ± SD, n = 3).

Samples	TL (g/100 g)	NL (g/100 g)	NL (%)	PL (g/100 g)	PL (%)
**CB**	1.42 ± 0.21	0.60 ± 0.13	42.25 ± 9.15	0.82 ± 0.13	57.74 ± 9.15
**LB**	1.25 ± 0.12	0.57 ± 0.04	45.60 ± 3.20	0.68 ± 0.14	54.40 ± 3.20
**IB**	1.44 ± 0.07	0.56 ± 0.07	38.89 ± 4.86	0.89 ± 0.05	61.81 ± 4.86
**HB**	1.15 ± 0.10	0.41 ± 0.04	35.65 ± 3.47	0.75 ± 0.13	65.22 ± 3.47

Abbreviations: TLs: total lipids; NLs: neutral lipids; PLs: polar lipids; CB, LB, IB and HB: the novel functional whole-grain breads containing low (LB), intermediate (IB) and high (HB) contents (concentrations) of the infused ACBPA-FGE-PL bioactives, versus control whole-grain bread (CB) that was not infused with ACBPA-FGE-PL bioactives.

**Table 4:** The fatty acid profile of the PL extracts for each apple pomace lipid extract-infused bread samples, expressed for each FA as percent composition of the total fatty acids in each sample assessed (mean ± standard deviation (SD); n = 3).

Fatty Acid Name	PL from CB	PL from LB	PL from IB	PL from HB
C8:0	0.11 ± 0.01	0.20 ± 0.02	0.04 ± 0.02	0.09 ± 0.03
C9:0	0.03 ± 0.01	ND	0.02 ± 0.01	ND
C10:0	0.01 ± 0.02	0.09 ± 0.02	0.1 ± 0.01	0.08 ± 0.01
C12:0	0.17 ± 0.02	0.20 ± 0.01	0.18 ± 0.02	0.2 ± 0.01
C13:0	0.004 ± 0.01	ND	0.01 ± 0.005	ND
C14:0	0.56 ± 0.06	0.66 ± 0.029	0.6 ± 0.01	0.6 ± 0.04
C15:0	0.103 ± 0.01	0.15 ± 0.01	0.12 ± 0.01	0.16 ± 0.007
C16:0	16.87 ± 0.35	19.63 ± 0.36	17.97 ± 0.46	19.21 ± 0.32
C16:1	2.08 ± 0.29	3.85 ± 0.39	3.88 ± 0.29	3.08 ± 0.24
C17:0	0.04 ± 0.03	0.08 ± 0.03	0.11 ± 0.05	0.1 ± 0.01
C18:0	0.36 ± 0.03	0.34 ± 0.09	0.31 ± 0.04	0.41 ± 0.03
C18:1 c9	22.85 ± 0.43	23.89 ± 0.36	22.16 ± 0.09	22.63 ± 0.28
C18:2 c9,12 (LA)	47.86 ± 0.48	43.46 ± 0.24	46.46 ± 0.55	45.50 ± 0.49
C18:3 c9,12,15 (ALA)	8.6 ± 0.21	7.14 ± 0.24	7.68 ± 0.52	7.58 ± 0.47
C18:4 c-3,6,9,12	ND	ND	ND	0.06 ± 0.01
C19:0	0.001 ± 0.00	0.0002 ± 0.0002	0.001 ± 0.001	ND
C20:0	0.08 ± 0.01	ND	ND	ND
C20:1	0.08 ± 0.02	0.14 ± 0.05	0.17 ± 0.04	0.17 ± 0.05
C20:2 c11,14	0.20 ± 0.002	0.17 ± 0.006	0.16 ± 0.001	0.15 ± 0.0001
C20:3	ND	0.01 ± 0.001	0.01 ± 0.002	ND
C22:5 c7,10,13,16,19 (DPA)	ND	0.004 ± 0.002	0.02 ± 0.01	0.003 ± 0.004
C22:6 c4,7,10,13,16,19 (DHA)	0.01 ± 0.00	ND	ND	ND
SFA	18.33 ± 0.56	21.34 ± 0.58	19.45 ± 0.64	20.82 ± 0.5
MUFA	25.01 ± 0.76	27.87 ± 0.8	26.21 ± 0.43	25.88 ± 0.6
PUFA	56.66 ± 0.69	50.79 ± 0.49	54.34 ± 1.09	53.31 ± 0.98
n6	48.05 ± 0.48	43.65 ± 0.24	46.64 ± 0.56	45.66 ± 0.5
n3	8.61 ± 0.21	7.14 ± 0.24	7.7 ± 0.53	7.65 ± 0.48
n6/n3	5.39 ± 0.16	6.12 ± 0.22	6.09 ± 0.48	5.96 ± 0.44

Abbreviations: n-3, omega-3 PUFA; n-6, omega-6 PUFA; PUFA, polyunsaturated fatty acids; MUFA, monounsaturated fatty acids; SFA, saturated fatty acids; ALA, alpha linolenic acid; LA, linoleic acid; EPA, eicosapentaenoic acid; DPA, docosapentaenoic acid; DHA, docosahexaenoic acid; ND, non-detectable. ND is defined as fatty acids detected with lower than 0.005% contribution to the overall fatty acid content.

**Table 5:** Total phenolic and carotenoid content of the FGE-ACBP-PL and the novel functional breads’ PLs.

Mean	HPC	LPC	TPC	TCC
	μg Quercetin eq/g Sample			ng β-Carotene eq/g Sample
ACBPA-FGE-PLs	1.86 ± 0.31	4.26 ± 0.35	6.11 ± 0.10	0.085 ± 0.022
ACBPB-FGE-PLs	4.11 ± 0.43 **	32.24 ± 0.94 **	36.35 ± 0.86 **	5.550 ± 0.171 **
ACBPC-FGE-PLs	1.43 ± 0.03	9.00 ± 0.46 *	10.43 ± 0.44 *	4.735 ± 0.763 **
PL from CB	0.783 ± 0.023	6.70 ± 0.60	7.49 ± 0.61	
PL from LB	1.34 ± 0.01 *	6.97 ± 1.50	8.31 ± 1.49	
PL from IB	1.14 ± 0.01 *	4.10 ± 0.56	5.23 ± 0.56	
PL from HB	1.41 ± 0.02 *	2.90 ± 0.25	4.30 ± 0.26	
Standard Quercetin (100 μg/mL)	2.02 ± 0.09	48.35 ± 1.13	50.37 ± 1.06	

* *p* < 0.05; ** *p* < 0.01; Abbreviations: PLs: polar lipids; FGE-ACBP-PL = food-grade extracted polar lipids from apple cider by-products; ACBPA, apple cider by-products of low-in-tannins Jonagold apple variety; ACBPB, apple cider by-products of medium-in-tannins Dabinett apple variety; ACBPC, apple cider by-products of high-in-tannins Aston Bitter apple variety; LB, IB, HB and CB = functional whole-grain breads containing low (LB), intermediate (IB) and high (HB) contents (concentrations) of the infused ACBPA-FGE-PL bioactives, versus control whole-grain bread (CB) that was not infused with ACBPA-FGE-PL bioactives; HPC = hydrophilic phenolic content; LPC = lipophilic phenolic content; TPC = total phenolic content.

**Table 6:** Anti-oxidant activities of the FGE-ACBP-PL and of the novel functional breads’ PLs.

Mean	FRAP-HAA	FRAP-LAA	FRAP-TAA	ABTS-HAA	ABTS-LAA	ABTS-TAA
	ug Trolox eq/g Sample			ug Trolox eq/g Sample		
ACBPA-FGE-PLs	662.69 ± 58.39	2450.2 ± 268.2	3112.9 ± 220.1	3757.2 ± 158.6	4367.7 ± 700.4	8124.9 ± 707.7
ACBPB-FGE-PLs	1289.1 ± 67.5	17,750 ± 1379	19,039 ± 1334	5959.6 ± 313.1	39,318 ± 1238	45,277 ± 1182
ACBPC-FGE-PLs	306.84 ± 29.98	4786.3 ± 346.6	5093.2 ± 358.1	852.63 ± 81.69	8239.7 ± 451.2	9092.4 ± 463.4
PLs from CB	114.06 ± 11.68	366.91 ± 66.16	480.97 ± 64.82	1054.6 ± 18.8	815.99 ± 36.8	1870.6 ± 42.2
PLs from LB	136.79 ± 4.08	639.72 ± 21.13 *	776.51 ± 20.18 *	993.20 ± 17.95	801.05 ± 27.15	1794.2 ± 36.7
PLs from IB	93.47 ± 6.03	713.03 ± 60.08 *	806.49 ± 59.17 *	643.42 ± 20.91	766.73 ± 67.97	1410.1 ± 81.7
PLs from HB	109.80 ± 7.90	823.94 ± 18.66 *	933.75 ± 16.77 *	689.47 ± 23.98	534.40 ± 22.86	1223.9 ± 18.2
Standard Quercetin	789.45 ± 67.90	33,625 ± 1260	34,414 ± 1310	2621.9 ± 103.4	42,440 ± 2729	45,061 ± 2717
Standard Gallic Acid	278,258 ± 18,430	1,587,042 ± 95,265	1,865,301 ± 108,547	587,228 ± 32,115	1,779,800 ± 42,845	2,367,028 ± 50,454

* *p* < 0.05; Abbreviations: PLs: polar lipids; ACBP-FGE-PL = food-grade extracted polar lipids from apple cider by-products; ACBPA, apple cider by-products of low-in-tannins Jonagold apple variety; ACBPB, apple cider by-products of medium-in-tannins Dabinett apple variety; ACBPC, apple cider by-products of high-in-tannins Aston Bitter apple variety; LB, IB, HB and CB= functional whole-grain breads containing low (LB), intermediate (IB) and high (HB) contents (concentrations) of the infused ACBPA-FGE-PL bioactives, versus control whole-grain bread (CB) that was not infused with ACBPA-FGE-PL bioactives; HAA = hydrophilic antioxidant activity; LAA = lipophilic antioxidant activity; TAA = total antioxidant activity.

## Data Availability

Access is restricted to protect proprietary information of the patent-invention. Available upon request, with permission from the PI of the project (AT).
